# Management of perivascular epithelioid cell tumor of the liver: A case report and review of the literature

**DOI:** 10.3892/ol.2013.1689

**Published:** 2013-11-19

**Authors:** DAREN LIU, DIKE SHI, YUANLIANG XU, LIPING CAO

**Affiliations:** Department of Surgery, Second Affiliated Hospital, Zhejiang University School of Medicine, Zhejiang, Hangzhou 310009, P.R. China

**Keywords:** perivascular epithelioid cell tumor, liver, neoplasm, human melanoma black-45

## Abstract

Perivascular epithelioid cell tumor (PEComa) is a rare tumor that originates from mesenchymal tissues. Cases of PEComa in the liver are extremely rare. The present study aimed to analyze the clinical features of PEComa in the liver and discuss its management. Here we report a 25-year-old female with multiple lesions of low density with poorly defined borders in the liver, as shown by a computed tomography (CT) scan. A partial hepatectomy was proceeded and PEComa was diagnosed by immunohistochemistry. No evidence of recurrence was observed during the one year follow-up. A total of 20 patients with hepatic PEComa, including one case from the present study and 19 cases that were reported in literature between June 2001 and December 2012, were reviewed and analyzed. The mean patient age was 43.4 years (range, 25–67 years) and the cases consisted of 18 female and two male patients. The tumor size ranged between 2.0×1.6 and 15.0×12.0 cm. Of the 20 patients, nine were asymptomatic and 11 had mild to significant complaints. Immunohistochemistry plays a key role in the diagnosis of PEComa. All the cases in this study were strongly positive for human melanoma black-45. A surgical resection is the gold standard for curative intent. All the patients underwent a surgical resection and none were administered perioperative chemotherapy or radiotherapy. In total, 13 of the 14 patients with follow-up information survived during the 8–36-month follow-up period and one patient succumbed due to recurrence two years after the surgery.

## Introduction

Perivascular epithelioid cells (PECs) were first proposed in 1992 by Bonetti *et al*(1) and classified by the World Health Organization in 2002 (2,3). The PEC tumor (PEComa) family is composed of epithelioid angiomyolipoma (AML), clear-cell ‘sugar’ tumors, lymphangioleiomyomatosis, clear-cell myomelanocytic tumors of the falciform ligament/ligamentum teres and rare clear-cell tumors of other anatomical sites (4–6). PEComa is mainly composed of eosinophilic and clear epithelioid cells, which are commonly arranged as small nests that are associated with variably-sized vessels (5–7). The predominant site of origin for PEComa is the uterus, but the tumor may also be found in the falciform ligaments, prostate and kidney. However, cases in the liver are extremely rare (2,8). Hepatic PEComa has a marked female predominance and possesses no specific symptoms (9). The diagnosis of PEComa is based on its pathological characteristics, including epithelioid cells without adipocytes or abnormal blood vessels, and on immunohistochemical evidence, including melanocytic and smooth muscle markers (8). Surgery is the only effective method to result in a long survival time (6,9).

In the present study, one case of hepatic PEComa is described. Furthermore, 19 cases from the literature, in which 11 patients were diagnosed with hepatic PEComa and eight with hepatic epithelioid angiomyolipoma, are reviewed.

## Case report

### Presentation and laboratory examinations

A 25-year-old female who presented with an abdominal mass, which was revealed by ultrasonography, was admitted to the Second Affiliated Hospital, Zhejiang University School of Medicine (Zhejiang, Hangzhou, China) in December 2011. The past history and physical examination were normal. The laboratory examinations revealed a slightly elevated level of carbohydrate antigen 19-9 (CA199; 38.8 kU/l; reference range, <37 kU/l). The levels of alanine aminotransferase (ALT; 7 U/l), aspartate aminotransferase (AST; 14 U/l), serum creatinine (SCr; 49 μmol/l), blood urea nitrogen (BUN; 3.80 mmol/l), α-fetoprotein (AFP; 2.2 μg/l) and carcinoembryonic antigen (CEA; 0.9 μg/l) were within the reference ranges. Hepatitis B and hepatitis C virus screenings were negative.

### Diagnostic techniques

**Figure 1:**
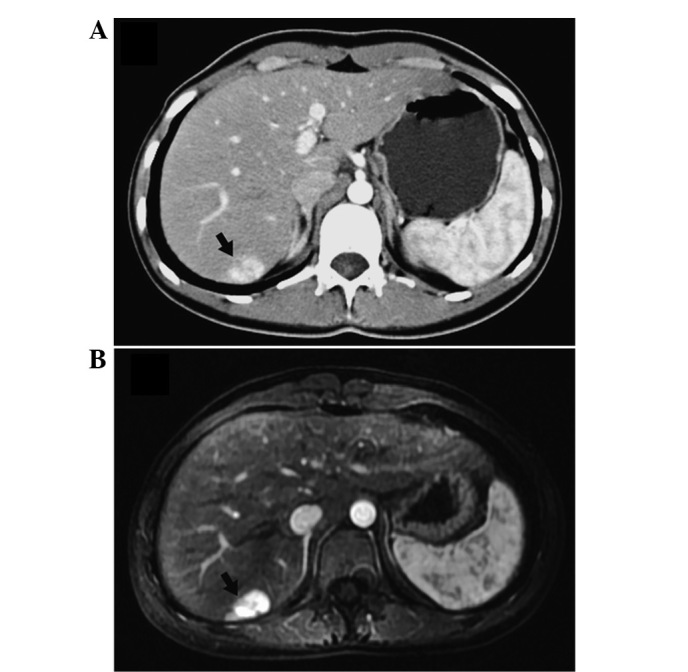
Imaging of hepatic PEComa. (A) Contrast-enhanced phase of CT reveals a poorly-defined mass with a significantly high intensity in segment VII of the liver. (B) The liver mass in segment VII revealed a strong enhancement in the contrast-enhanced phase of the MRI. PEComa, perivascular epithelioid cell tumor; CT, computed tomography; MRI, magnetic resonance imaging.

A plain computed tomography (CT) scan demonstrated multiple lesions of low density with poorly-defined borders in hepatic segments III (0.5×0.5 cm), IV (5.4×5.5 cm) and VII (1.8×1.5 cm). The delayed phase showed mild intensity lesions. However, the intensity of the lesions increased significantly in the contrast-enhanced phase (Fig. 1A). A liver magnetic resonance imaging (MRI) scan revealed that the lesions had a medium signal intensity on the T1-weighted image and a slightly high signal on the T2-weighted image. The enhancement of intensity of the lesions was also observed in the contrast-enhanced phase of MRI (Fig. 1B).

### Treatment

During the laparotomy, three well-encapsulated tumors located in segment III, IV and VII were identified. No portal or inferior vena cava vein invasion or distant metastasis was observed. A partial hepatectomy of the liver neoplasms was performed using the Pringle maneuver.

### Pathology and immunohistochemical analysis

**Figure 2:**
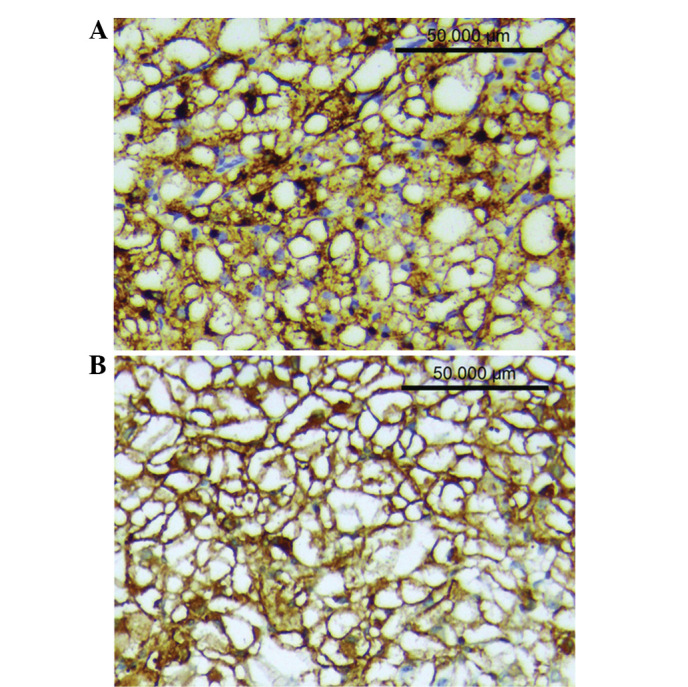
Immunohistochemical results of hepatic PEComa (x200). (A) Tumor cells showing strong and diffuse positive staining for HMB-45 (+++). (B) Tumor cells showing strong and diffuse positive staining for SMA (+++). PEComa, perivascular epithelioid cell tumor; HMB-45, human melanoma black-45; SMA, smooth muscle actin.

The masses in segments III and IV were pathologically identified as hemangioma, while the mass in segment VII was revealed to be composed of polygonal morphology cells, which are similar to epithelial cells. However, the latter mass did not contain lipocytes or abnormal blood vessels. Immunohistochemistry further revealed that the mass in segment VII was strongly positive for human melanoma black-45 (HMB-45; +++; Fig. 2A), smooth muscle actin (SMA; +++; Fig. 2B) and vimentin (++). The mass was positive for CD34, but negative for S-100, creatine kinase (CK), epithelial membrane antigen (EMA), desmin, AFP and Ki67. A diagnosis of PEComa of the liver was confirmed based on the immunohistochemical analysis. The patient recovered well and was discharged one week after surgery. No evidence of recurrence was observed during one year of follow-up.

### Study approval

Approval for this study was obtained from the ethics committee of Zhejiang University and informed consent was provided by the patient.

### Literature review

#### Identification of patients with hepatic PEComa

The Chinese BioMedical Literature Database, the China Hospital Knowledge Database and the Wanfangbase were searched between January 2001 and December 2012. Repeated studies were carefully screened and rejected from further analysis to avoid over-representation. The final diagnosis of each case was confirmed using pathological and immunohistochemical results. The data on the clinical features, tumor characteristics, pre-operative imaging and outcome of treatment of 19 cases were collected and analyzed along with the data of the present case.

#### Presentation

The mean patient age was 43.4 years (range, 25–67 years) in the 20 patients. PEComa was shown to have a marked female predominance (18 females and two males; Table I). The chief presenting complaints were abdominal pain (5/20), abdominal discomfort (5/20) and abdominal distension (1/20). Of the 20 patients, nine were asymptomatic and discovered the mass incidentally during a physical examination. The majority of the tumors were localized in the right lobe (16/20) of the liver. The tumor size ranged between 2.0×1.6 cm and 15.0×12.0 cm. Of the 20 tumors that were analyzed, seven were >5 cm in size at the time of presentation.

#### Pathological findings and immunohistochemistry

Histological examination revealed that the tumor was highly cellular, with large round or polygonal cells with abundant cytoplasm and clear cell boundaries. Immunohistochemistry revealed that the tumor cells in all cases were positive for HMB-45 (20/20) and melan-A (9/9). The cells were positive for SMA (14/16), vimentin (12/13) and S-100 (7/13) in the majority of the cases (Table II).

#### Treatment

All 20 patients underwent surgery following admission. Three patients were treated with a hemihepatectomy, three with a segmentectomy and 10 with a partial hepatectomy of the liver neoplasms. However, no patients were administered chemotherapy or radiotherapy.

#### Follow-up

The follow-up data was available for 14 patients and the follow-up time ranged between 8 and 36 months. One patient succumbed due to recurrence at two years post-surgery and the others survived without recurrence or metastasis.

## Discussion

The pathogenesis of PEComa, although discussed in previous studies, remains controversial. Kenerson *et al*(10) demonstrated that tuberous sclerosis complex 1/2 (TSC1/2) inactivation and mTOR hyperactivation were present in non-TSC AMLs and extrarenal PEComas using immunohistochemistry and western blot analysis. In particular, mTOR hyperactivation may be studied in such lesions using immunohistochemical detection of p70S6K, which is a marker of mTOR activity (6). Bing *et al*(11) also showed that epithelioid AMLs harbored p53 mutations in certain cases.

Hepatic PEComa occurs most commonly in females (7), and symptoms of hepatic PEComa usually show no specificity. In the present study, 11 out of 20 patients with hepatic PEComa had mild to significant non-specific complaints. The remaining nine patients were asymptomatic. The majority of the patients (16/20) had solitary lesions in the right lobe, which was consistent with a previous study by Parfitt *et al*(7).

Ultrasonograpy, CT and MRI are most frequently employed for the pre-operative diagnosis of PEComa. Previous studies have considered hypervascularity and arteriovenous connections to be a feature of PEComa in contrast-enhanced CT (8,12,13). An MRI scan revealed that the PEComas were significantly and heterogeneously enhanced in the arterial phase, but less enhanced in the portal venous and delayed phases (14,15), which may also effectively rule out a diagnosis of hepatocellular carcinoma with a fibrotic capsule (15). Another commonly used diagnostic method is contrast-enhanced ultrasonography, which possesses the features of an early influx of the contrast agent into the tumor and a rapid drainage of arterial blood to the veins (15). However, the accuracy of pre-operative diagnosis is low, partly as a result of the variable imaging appearances due to the varying proportion of the components, including the smooth muscle cells, adipose tissue and vessels, and the rarity of the tumor. In the present case, three lesions of the liver shared similar imaging features, but only one lesion was confirmed to be a PEComa. In the cases that were reviewed in this study, three patients were pre-operatively diagnosed with focal nodular hyperplasia (including the present case), four patients were diagnosed with hepatic carcinoma and 12 patients could not be differentiated.

The final diagnosis of PEComa depends on the pathology and immunohistochemistry. Martignoni *et al*(6) defined PEComa as a tumor that is composed of solely of cells with an epithelioid appearance, which are closely associated with dilated vascular channels and contain clear eosinophilic cytoplasms, but do not contain lipocytes or disordered blood vessels. Almost all the PEComas were identified to be strongly positive for melanocytic markers (HMB-45 and/or melan-A) and smooth muscle markers (SMA and/or desmin) (2,5,16). In the present study, the tumor cells of the 14 patients were positive for HMB-45 and SMA, but in the case reported by Zou *et al*(28), only HMB-45 and melan-A were positive and SMA was not stained.

PEComa has been reported to exhibit benign behavior in the majority of the literature. Since the first case of malignant hepatic PEComa presented by Dalle *et al*(17), several malignant cases have been reported (7,17,18). In 2005, Folpe *et al*(16) reviewed 26 cases of soft tissue and gynecological origins and raised seven criteria to evaluate the malignancy of PEComa: i) A tumor size of >5 cm; ii) infiltration into the surrounding normal tissue; iii) a high nuclear grade; iv) hypercellularity; v) high mitotic activity >1/50 high-power field); vi) coagulative necrosis of the tumor; and vii) vascular invasion. A malignant PEComa is considered to have two or more of the features that are listed. Tumors with nuclear pleomorphisms, multinucleated giant cells only or those of >5 cm in size are considered as neoplasms of uncertain malignant potential. In the present study, one case of malignant hepatic PEComa was also reviewed in which the patient was identified to have a recurrence two years after the surgery.

Surgical resection with an adequate margin remains the gold standard for the treatment of hepatic PEComa (6,9,14), particularly in malignant cases. In the present study, all patients underwent surgical treatment. The malignant patient underwent a hemihepatectomy due to the enormous tumor size. Hemihepatectomies were also performed in another two patients, who were misdiagnosed with hepatocellular carcinoma prior to the surgery. Generally, chemotherapy and radiotherapy does not indicate an improved survival time (6). However, several studies have shown promising treatment results, including a study of rapamycin, which is an inhibitor for mTOR. Rapamycin had a positive effect on renal angiomyolipoma (5). If rapamycin is able to yield the same effect on other PEComas, this would provide the rationale for lesions that are composed of PECs. Of the 14 patients in the present study, 13 were alive during the 8–36-month follow-up period and one succumbed due to recurrence. However, Parfitt *et al*(7) noted that PEComa may also demonstrate recurrence following a long period of time (nine years). Thus, the prognosis of PEComa remains unpredictable, and it is necessary to perform long-term follow-up studies for every case.

In conclusion, the diagnosis of hepatic PEComa depends on the pathological observations. Surgical resection of the tumor appears to be necessary for a cure, particularly for malignant tumors. Although adjuvant chemotherapy or radiotherapy are not significant in the treatment, several studies that have used drugs to treat PEComa have shown promising results. In the present study, the tumors were mostly benign and the prognosis following surgical resection was good.

## Figures and Tables

**Table I:** Clinical data of 20 cases.

First author, year (ref.)	Gender/age, years	Symptom	Size, cm	Location, lobe
Present case	F/25	None	2.5×1.0	Left
Qiu *et al*, 2008 (19)	F/67	Abdominal pain	15.0×12.0	Right
Deng *et al*, 2011 (20)	M/66	None	3.0×3.5	Left
He *et al*, 2011 (21)	F/35	None	3.5×3.0	Right
Han *et al*, 2008 (22)	M/44	None	2.0×1.6	Right
Li *et al*, 2007 (23)	F/56	Abdominal distension	5.0×4.0	Left
Chen and Li, 2009 (24)	F/37	Abdominal pain	5.0×4.0	Right
Zhang and Wang, 2012 (25)	F/55	None	3.0×3.0	Right
Chen, 2009 (26)	F/36	Abdominal pain	7.0×5.0	Right
Chen, 2009 (26)	F/45	Abdominal discomfort	5.5×4.0	Right
Liu *et al*, 2010 (27)	F/32	None	5.5×5.5	Right
Zou *et al*, 2011 (28)	F/54	Abdominal discomfort	6.0×5.0	Right
Xu *et al*, 2001 (29)	F/35	None	2.0×2.0	Right
Lin *et al*, 2002 (30)	F/30	Abdominal discomfort	3.6×3.1	Right
Liu *et al*, 2008 (31)	F/31	Abdominal pain	8.0×6.0	Right
Zhu *et al*, 2010 (32)	F/26	None	5.0×3.0	Right
Gao *et al*, 2012 (33)	F/59	Abdominal pain	6.0×5.0	Right
Wang *et al*, 2007 (34)	F/46	None	4.0×4.0	Right
Shi *et al*, 2010 (35)	F/41	Abdominal discomfort	5.5×4.0	Left
Shi *et al*, 2010 (35)	F/48	Abdominal discomfort	8.0×6.0	Right

M, male; F, female.

**Table II:** Results of the immunohistochemistry.

Immunohistochemistry	Frenquency, % (Positive cases/Total cases)
HMB-45	100 (20/20)
SMA	88 (14/16)
Melan-A	100 (9/9)
Vimentin	92 (12/13)
S-100	54 (7/13)
Desmin	40 (2/5)
CD34	45 (5/11)
AFP	0 (0/10)
EMA	0 (0/9)

HMB-45, human melanoma black-45; SMA, smooth muscle actin; AFP, α-fetoprotein; EMA, epithelial membrane antigen.
